# A New Approach to Dengue Fatal Cases Diagnosis: NS1 Antigen Capture in Tissues

**DOI:** 10.1371/journal.pntd.0001147

**Published:** 2011-05-03

**Authors:** Monique da Rocha Queiroz Lima, Rita Maria Ribeiro Nogueira, Hermann Gonçalves Schatzmayr, Ana Maria Bispo de Filippis, Daniel Limonta, Flavia Barreto dos Santos

**Affiliations:** 1 Flavivirus Laboratory, Oswaldo Cruz Institute, Rio de Janeiro, Brazil; 2 National Reference Laboratory for Dengue, Virology Department, PAHO/WHO Collaborating Center for the Study of Dengue and Its Vector, Pedro Kourí Institute of Tropical Medicine, Havana, Cuba; University of Rhode Island, United States of America

## Abstract

***Abstract/Background*:**

Dengue is the most important arthropod borne viral disease worldwide in terms of morbidity and mortality and is caused by any of the four serotypes of dengue virus (DENV-1 to 4). Brazil is responsible for approximately 80% of dengue cases in the Americas, and since the introduction of dengue in 1986, a total of 5,944,270 cases have been reported including 21,596 dengue hemorrhagic fever and 874 fatal cases. DENV can infect many cell types and cause diverse clinical and pathological effects. The goal of the study was to investigate the usefulness of NS1 capture tests as an alternative tool to detect DENV in tissue specimens from previously confirmed dengue fatal cases (*n* = 23) that occurred in 2002 in Brazil.

***Methodology/Principal Findings*:**

A total of 74 tissue specimens were available: liver (*n* = 23), lung (*n* = 14), kidney (*n* = 04), brain (*n* = 10), heart (*n* = 02), skin (*n* = 01), spleen (*n* = 15), thymus (*n* = 03) and lymph nodes (*n* = 02). We evaluated three tests for NS1 antigen capture: first generation Dengue Early ELISA (PanBio Diagnostics), Platelia NS1 (BioRad Laboratories) and the rapid test NS1 Ag Strip (BioRad Laboratories). The overall dengue fatal case diagnosis based on the tissues analyzed by Dengue Early ELISA, Platelia NS1 and the NS1 Ag Strip was 34.7% (08/23), 60.8% (14/23) and 91.3% (21/23), respectively. The Dengue Early ELISA detected NS1 in 22.9% (17/74) of the specimens analyzed and the Platelia NS1 in 45.9% (34/74). The highest sensitivity (78.3%; 58/74) was achieved by the NS1 Ag Strip, and the differences in the sensitivities were statistically significant (*p<0.05*). The NS1 Ag Strip was the most sensitive in liver (91.3%; 21/23), lung (71.4%; 10/14), kidney (100%; 4/4), brain (80%; 8/10), spleen (66.6%, 10/15) and thymus (100%, 3/3) when compared to the other two ELISA assays.

***Conclusions/Significance*:**

This study shows the DENV NS1 capture assay as a rapid and valuable approach to postmortem dengue confirmation. With an increasing number of DHF and fatal cases, the availability of new approaches useful for cases confirmation plays an important tool for the disease surveillance.

## Author Summary

Dengue manifestations may vary from asymptomatic to potentially fatal complications. With an increasing number of Dengue Hemorrhagic fever (DHF) and fatal cases, the availability of new approaches useful for cases confirmation plays an important role for the disease surveillance. The diagnosis of fatal cases in frozen and fixed tissues from autopsies can be determined by techniques such as viral RT-PCR, in situ hybridization, viral proteins detection by immunohistochemistry and NS3 specific immunostaining. We aimed to assess for the first time the usefulness of NS1 capture tests as a diagnostic technique to demonstrate DENV antigens in human tissue specimens. The highest sensitivity was obtained by a rapid ICT which was also the most sensitive in liver, lung, kidney, brain, spleen and thymus. Despite a number of studies demonstrating the usefulness of DENV NS1 antigen detection by different ELISAs in plasma and/or sera of dengue patients, no research has been done previously to demonstrate NS1 presence in tissues of fatal dengue cases. Moreover, the application of NS1 kits to demonstrate the presence of DENV may provide a better understanding of viral tropism in fatal cases and may be useful for studies of pathogenesis in vivo and in experimental animals.

## Introduction

Dengue virus (DENV) infection is recognized as one of the most important mosquito borne human infections in the 21^th^ century. The new estimates of the burden of dengue has increased with 2.5 billion people worldwide at risk of contracting the disease, 55% of world population, and an estimated 70–500 million of dengue infections occurring annually in 100 endemic countries that includes approximately 22, 000 fatal cases [1] . Dengue can cause a mild disease known as dengue fever and more severe and potentially fatal clinical forms, the Dengue Hemorrhagic Fever (DHF) and Dengue Shock Syndrome (DSS) [2].

In Brazil, the disease is an important public health problem associated with explosive epidemics and since DENV introduction in 1986 [3], a total of 5,944,270 cases were reported including 21,596 DHF and 874 fatal cases.

Currently, laboratorial diagnosis of dengue suspected cases is based on virus isolation in mosquito cell cultures, detection of viral RNA and DENV specific antibodies in serum or plasma [4]. However, a number of studies have shown previously that the DENV nonstructural 1 (NS1) antigen, a highly conserved glycoprotein produced in both membrane-associated and secreted forms, is abundant in the serum of patients in the early phase of infection [5], [6], [7], [8], [9] and it is useful in the diagnosis of dengue infection [8], [10], [11], [12], [13] . Furthermore, an evaluation of the three NS1 tests for early diagnosis of dengue in Brazil was performed previously [14].

On the other hand, the virological diagnosis in tissues specimens from dengue fatal cases shows a more complex scenario. The presence of DENV in frozen and fixed tissues from autopsies can be determined by viral RNA detection by RT-PCR [15], [16] and in situ hybridization [17], and/or viral proteins detection by immunohistochemistry [17], [18], [19], [20] and NS3 specific immunostaining [21] in tissues such as liver, spleen, brain, lung, lymph node, thymus, kidney, heart, bone marrow and skin. DENV was previously detected by immunohistochemistry, conventional RT-PCR and Real-Time RT- PCR in a number of Brazilian human tissues [22].

Here, we aimed to assess for the first time the usefulness of NS1 capture tests as a diagnostic technique to demonstrate DENV antigens in human tissue specimens. In this retrospective study was used tissues homogenates from dengue fatal cases occurred in Brazil in 2002.

## Materials and Methods

### Ethics Statement

The specimens analyzed in this study belong to a previously-gathered collection from the Laboratory of Flavivirus, IOC/FIOCRUZ from an ongoing Project approved by resolution number CSN196/96 from the Oswaldo Cruz Foundation Ethical Committee in Research (CEP 274/05), Ministry of Health, Brazil.

### Clinical samples

The human tissues analyzed in this study were obtained from the collection of the Laboratory of Flavivirus at Oswaldo Cruz Institute, FIOCRUZ, Brazil, from the epidemic occurred in 2002. A total of 74 tissue samples were available from 23 fatal cases: liver (*n* = 23), lung (*n* = 14), kidney (*n* = 04), brain (*n* = 10), heart (*n* = 02), skin (*n* = 01), spleen (*n* = 15), thymus (*n* = 03) and lymph nodes (*n = *02). Tissues sample collections were performed up to 12 hours (median 6 hours) post-mortem according to the Brazilian Ministry of Health necropsy protocol recommendations and stored at −70°C until used.

### Case confirmation methodology

As a laboratorial routine, all suspected dengue fatal cases are submitted to all diagnostic methods available in the laboratory to confirm dengue infection: virus isolation [23], [24], RT-PCR [25], Real Time RT-PCR [26] and also immunohistochemistry when formalin-fixed [19], paraffin-embedded tissues [19] are available.

### Virus isolation

Virus isolation was performed by inoculation into C6/36 *Aedes albopictus* cell line [23] and isolates were identified by indirect fluorescent antibody test (IFAT) using serotype-specific monoclonal antibodies [24].

### RT–PCR

RT-PCR for detecting and typing DENV was performed as described previously [25]. Briefly, consensus primers were used to anneal to any of the four DENV types and amplify a 511-bp product in a reverse transcriptase-polymerase reaction. A cDNA copy of a portion of the viral genome was produced in a reverse transcriptase reaction. After a second round of amplification (nested PCR) with type-specific primers, DNA products of unique sizes for each dengue virus serotype were generated.

### Real-time Reverse Transcriptase PCR (TaqMan) assay

One-step real-time RT-PCR assays were performed in the ABI Prism 7000 Sequence Detection System (SDS) (Applied Biosystems, Foster City, CA) as described previously [26]. Briefly, samples were assayed in a 30 µL reaction mixture containing the extracted RNA, 40× Multiscribe enzyme plus RNAse inhibitor, TaqMan 2× Universal PCR Master Mix (Applied Biosystems, Foster City, CA) and each specific primer and fluorogenic probe labeled at the 5′ end with 5-carboxyfluorescein (FAM) reporter dye and at the 3′ end with 6-carboxy-*N*,*N*,*N*′,*N*′-tetramethylrhodamine (TAMRA) quencher fluorophore. Amplification and real-time detection consisted of a reverse transcription at 45°C for 30 min followed by one step at 95°C for 10 min and 45 cycles at 95°C for 15 s and 60°C for 1 min.

### Immunohistochemistry procedure

The immunohistochemistry procedure was performed as described previously [19]. Briefly, sections of formalin-fixed, paraffin-embedded tissues were processed by the avidin biotin complex (ABC) method according to the manufacturer's protocol (Vectastain AEC Kit, Vector Laboratories, Inc. Burlingame, CA, USA). Monoclonal antibodies for DENV-1, -2, and -3 were directed against the E protein. Positive and negative controls were included.

### Tissue treatment

Frozen fragments of human tissue (1–2 g) kept at −70°C were ground and centrifuged as previously described [22]. Briefly, by using sterile tweezers and scissors a tissue fragment of approximately 1 cm^3^ was cut, transferred and ground by mortar and pestle procedure in 1.5 ml of Leibovitz-15 medium (Sigma), pH 7.0–7.4 and 3% sodium penicillin/ streptomycin sulfate. The ground suspension was transferred to a 15 ml conical tube, incubated at 4°C for 60 minutes and centrifuged (10,000 rpm at 4°C, for 15 min). The clear supernatant obtained was transferred to a sterile 2.0 mL cryotube and stored at −70°C until used. The supernatant used for virus isolation and RNA extraction previously [22] was used in the present study for all NS1 antigen capture tests.

### Control tissues

Three liver tissues available from cases negative for DENV infection by using all the methods described above were used as negative controls. Liver (*n* = 05), lung (*n* = 01), spleen (*n* = 03) and brain (*n = *01) tissues from confirmed yellow fever fatal cases were used to test the cross-reactivity of the NS1 assays. Unfixed frozen control tissues were prepared and tested in the same manner as for the dengue positive tissues.

### NS1 antigen capture methods

#### Dengue Early ELISA, first generation (PanBio Diagnostics)

The test (Panbio Diagnostics, Brisbane, Australia) is based on a one-step sandwich format microplate enzyme immunoassay to detect DENV NS1 antigen. Briefly, the specimens were allowed to thaw to laboratory ambient temperature (21–22°C). One hundred microliters of the sample and controls were pipetted into their respective microwells and incubated for 60 min at 37°C. After a six-times washing step, 100 µL of HRP conjugate anti-NS1 MAb were pipetted into each well and plate was incubated for 60 min at 37°C. After a six-times washing step, 100 µL of substrate were pippeted into each well and plate was incubated for 10 min at room temperature in the dark. The presence of immune-complex was demonstrated by a color development and the enzymatic reaction was stopped by adding 100 µL of 1 M H_3_PO_4_. The optical density (OD) reading was taken with a spectrophotometer at a wavelength of 450–620 nm and the amount of NS1 antigen present was determined by comparing the OD of the sample tested to the OD of the cut-off control. Results were calculated as “Panbio units” with results <9.0, 9.0–11.0, and ≥11.0 defined as negative, inconclusive, and positive, respectively. Inconclusive samples were re-tested to confirm the result.

#### Platelia Dengue NS1 Ag ELISA (BioRad Laboratories)

The test system (Platelia Dengue NS1 Ag ELISA, BioRad Laboratories, France) is based on a one-step sandwich format microplate enzyme immunoassay to detect DENV NS1 antigen in human serum or plasma. The test uses murine monoclonal antibody for capture and revelation. If NS1 antigen is present in the sample, an immune-complex MAb-NS1-MAb/peroxidase will be formed. Briefly, the specimens were allowed to thaw to laboratory ambient temperature (21–22°C). Sample diluent (50 µl), respective samples and controls (50 µl each) and 100 µl of diluted conjugate were incubated for 90 min at 37°C within the respective microplate wells coated with purified mouse anti- NS1 monospecific antibodies. After a six-times washing step, 160 µl of substrate was added into each well and incubated for 30 min at room temperature in the dark. The presence of immune-complex was demonstrated by a color development and the enzymatic reaction was stopped by adding 100 µl of 1 N H_2_SO_4_. The OD reading was taken with a spectrophotometer at a wavelength of 450–620 nm and the amount of NS1 antigen present was determined by comparing the OD of the sample to the OD of the cut-off control.

#### Dengue NS1 Ag STRIP (Bio-Rad Laboratories)

Dengue NS1 Ag STRIP (BioRad Laboratories, France) is an immunochromatographic test (ICT) for the rapid detection of NS1 antigen. Briefly, one drop of migration buffer was added to 50 µL of sample specimen in a tube and a strip was placed in the tube. The strip has two lines: a control line (C) (‘biotin – gold colloidal particles coated with streptavidin’ complex) and a test line (T) (‘monoclonal anti-NS1 antibodies (mAb) – NS1 Ag – gold colloidal particles coated with anti-NS1 mAb’ complex). The appearance of the T and C lines after a migration time of 15 minutes (min) indicates a positive result. The appearance of the C line alone indicates a negative result. If the C line is not present, the test is considered invalid and is repeated. It is recommended that strips giving ambiguous (faint color at the T line) or negative results are put back in the tube after the initial reading and left for a further 15 min for re-evaluation.

### Statistical analysis

The derived data was tabulated in appropriate worksheets using the Microsoft Excel programmer and evaluated by chi-square test using the Epi Info 6 (Center for Disease Control and Prevention, Atlanta) for any statistical significant association.

## Results

Tissues from 23 fatal cases (11 males and 12 females with an age range of 20–64 y (mean = 36 y) were submitted to NS1 antigen capture tests as a novel approach for dengue fatal case diagnosis. All cases were submitted to routine diagnosis of fatal dengue available in the laboratory: virus isolation, RT-PCR, Real Time RT-PCR and immunohistochemistry when formalin-fixed paraffin-embedded tissues were also available. From our previous investigation [22], it was shown that DENV-3 could be identified by virus isolation and/or RT-PCR in 47.8% (11/23) of the cases and viral RNA could be detected in 91.3% (21/23) of the cases by Real-time RT-PCR. Viral antigen was detected in 63.1% (12/19) of the specimens by immunohistochemistry. Fatal case # 9 was confirmed as dengue case by positive results by Real-time RT-PCR in CSF and blood, specimens not included in this study (Table 1).

**Table 1:** NS1 antigen capture tests analysis in tissues from dengue fatal cases (*n = *23).

Case information						Case confirmation methods (Araujo et al., 2009)				NS1 antigen capture tests analyzed (this study)		
Fatal Case	Gender	Age	Days of illness	Fresh tissues available	Immune response (IgG titer)	Virus Isolation Serotype (tissue)	RT-PCR Serotype (tissue)	Real-time RT-PCR (tissue)	Immuno Histo chemistry (tissue)	Early ELISA (tissue)	Platelia (tissue)	NS1 Ag STRIP (tissue)
**1**	F	62	NA	Liver, lung, kidney, brain, spleen	NA	−	+ DENV-3 (liver, kidney, brain)	+ (liver, lung, brain, spleen)	−	−	−	+ (liver, kidney, brain)
**2**	M	55	4	Liver, lung, brain, spleen	P (<40)	−	−	+ (lung, brain)	−	+ (liver, lung,brain, spleen)	−	+ (liver, lung,brain, spleen)
**3**	F	39	3	Liver, skin	P (<40)	−	+ DENV-3 (liver, skin)	−	+ (liver)	−	+ (liver, skin)	+ (liver, skin)
**4**	M	26	16	Liver, lung	NA	−	−	+ (liver, lung)	−	−	−	+ (liver, lung)
**5**	F	43	NA	Liver, spleen	NA	+ DENV-3 (liver)	+ DENV-3 (liver)	+ (liver)	NA	+ (liver)	+ (liver)	+ (liver)
**6**	M	26	NA	Liver, lung, brain, thymus, lymph nodes	NA	−	−	+ (liver, lung, brain)	NA	+ (liver)	−	+ (liver, lung,brain, thymus)
**7**	M	NA	NA	Liver, lung, brain, spleen, thymus, lymph nodes	NA	−	−	+ (liver)	NA	+ (liver, lung, spleen, thymus, lymph nodes)	−)	+ (liver,brain, spleen, thymus)
**8**	M	49	3	Liver, lung, brain, spleen	NA	−	−	+ (lung)	+ (liver, lung,brain, spleen)	+ (liver,lung, spleen)	+ Liver, lung, brain, spleen)	−
**9**	M	55	6	Liver, lung	P (1/160)	−	−	−	−	−	+ (liver, lung)	+ (liver, lung)
**10**	F	NA	NA	Liver, lung, spleen	NA	−	+ DENV-3 (liver, lung)	+ (liver, lung)	+ (liver, spleen)	+ (lung)	+ (liver, lung, spleen)	+ (liver, lung, spleen)
**11**	M	20	NA	Liver, kidney, heart, spleen	NA	−	+ DENV-3 (liver, spleen)	+ (kidney, heart, spleen)	+ (kidney)	−	−	+ (liver, kidney)
**12**	M	63	NA	Liver, brain, spleen, thymus	NA	−	−	+ (liver, brain)	NA	+ (brain, spleen)	−	+ (liver, brain, spleen, thymus)
**13**	F	41	6	Liver, lung, brain, spleen	NA	−	−	+ (spleen)	−	−	+ (liver, lung, spleen)	+ (liver, lung, spleen)
**14**	F	38	7	Liver	NA	−	+ DENV-3 (liver)	+ (liver)	+ (liver)	−	+ (liver)	+ (liver)
**15**	F	21	3	Liver, lung, brain, spleen	P (<40)	+ DENV-3 (liver)	+ DENV-3 (liver, lung, brain, spleen)	+ (liver, brain, spleen)	+ (liver, lung, spleen)	−	+ (liver, lung, brain, spleen)	+ (liver, lung, brain, spleen)
**16**	M	33	4	Liver, lung brain, spleen	P (<40)	−	+ DENV-3 (lung)	+ (lung, brain, spleen)	+ (liver, brain)	−	−	+ (lung, brain)
**17**	F	51	NA	Liver, spleen	P (<40)	−	−	+ (liver, spleen)	+ (liver, spleen)	+ (liver)	+ (liver, spleen)	+ (liver, spleen)
**18**	F	38	7	Liver	NA	−	−	+ (liver)	+ (liver)	−	+ (liver)	+ (liver)
**19**	F	30	NA	Liver	P (<40)	−	−	+ (liver)	+ (liver)	−	−	−
**20**	F	42	NA	Liver, lung, kidney, heart, spleen	NA	−	+ DENV-3 (liver, lung,, heart, spleen)	+ (liver, lung, kidney, spleen)	NA	+ (heart)	+ (liver, lung, kidney, heart, spleen)	+ (liver, lung, kidney, heart, spleen)
**21**	M	51	NA	Liver	NA	−	+ DENV-3 (liver)	−	−	−	+ (liver)	+ (liver)
**22**	M	64	NA	Liver, spleen	NA	−	−	+ (liver, spleen)	+ (liver)	−	+ (liver, spleen)	+ (liver, spleen)
**23**	F	NA	NA	Liver,lung, kidney, brain, spleen	NA	−	+ DENV-3 (liver, lung, kidney, brain)	+ (liver, kidney)	−	+ (lung)	+ (liver,lung,kidney, brain, spleen)	+ (liver,lung,kidney, brain, spleen)
Total*						2/23 (8.6)	11/23 (47.8)	20/23 (86.9)	11/19 (57.8)	8/23 (34.7)	14/23 (60.8)	21/23 (91.3)

The overall dengue fatal case confirmation based on the tissues analyzed by Early ELISA (Panbio), Platelia NS1 (Biorad) and the NS1 Ag Strip (Biorad) was 34.7% (08/23), 60.8% (14/23) and 91.3% (21/23), respectively (Table 1).

The NS1 antigen capture tests performance according to the different tissues available is shown on Table 2. The Early ELISA detected NS1 in 22.9% (17/74) of the tissues specimens analyzed and the Platelia NS1, 45.9% (34/74). The highest sensitivity (78.3%; 58/74) was by the NS1 Ag Strip, and the differences in the sensitivities were statistically significant (*p*<0.05). The NS1 Ag Strip was the most sensitive in liver (91.3%, 21/23), lung (71.4%, 10/14), kidney (100%, 4/4), brain (80%, 8/10), spleen (66.6%, 10/15) and thymus (100%, 3/3) when compared to the other two assays. Lymph node from one case was positive only by the Early ELISA. The only skin sample in the study was positive by both Biorad tests.

**Table 2:** NS1 antigen capture tests applied to tissues (*n = *74) from confirmed dengue fatal cases (*n = *23).

Tissues analyzed	Confirmation methods (Araujo et al., 2009) Positive/Tested (%)				NS1 antigen capture tests analyzed (this study) Positive/Tested (%)		
	Virus isolation	RT-PCR	Real-time RT-PCR	Immuno Histo Chemistry	Early ELISA	Platelia	NS1 Ag STRIP
**Liver (*n = *23)**	2/22 (9)	10/23 (43.4)	16/23 (69.5)	12/17 (70.5)	6/23 (26)	14/23 (60.8)	21/23 (91.3)
**Lung (*n = *14)**	0/14	5/14 (35.7)	8/14 (57.1)	3/10 (30)	4/14 (28.5)	5/14 (35.7)	10/14 (71.4)
**Kidney (*n = *04)**	0/4	2/4 (50)	3/4 (75)	1/2 (50)	0/4	2/4 (50)	4/4 (100)
**Brain (*n = *10)**	0/10	3/10 (30.0)	6/10 (60.0)	2/6 (33.3)	2/10 (20.0)	3/10 (30)	8/10 (80.0)
**Heart (*n = *02)**	0/2	½ (50)	½ (50)	0/1	0/2	½ (50)	½ (50)
**Skin (*n = *01)**	ND	1/1 (100)	ND	0/1	0/1	1/1 (100)	1/1 (100)
**Spleen (*n = *15)**	0/14	3/14 (21.4)	8/15 (53.3)	5/10 (50)	3/15 (20)	8/15 (53.3)	10/15 (66.6)
**Thymus (*n = *03)**	0/3	0/3	0/3	NA	1/3 (33.3)	0/3	3/3 (100)
**Lymph nodes (*n = *02)**	0/2	0/2	0/2	NA	1/2 (50)	0/2	0/2
**Total (*n = *74)**	**2/71 (2.8)**	**25/73 (34.2)**	**42/73 (57.5)**	**23/47 (48.9)**	**17/74 (22.9)**	**34/74 (45.9)**	**58/74 (78.3)**

*Total positive /total tested (%), ND: not done, NA: not available.

Table 3 shows the NS1 antigen capture contribution, independently of the test used in the different tissues analyzed per case. The overall sensitivity of this new approach in confirming the fatal cases was 87.0%. Only fatal case number 19, with only a liver tissue sample, was not confirmed by any of the NS1 tests used while 22 out of 23 cases evaluated had at least one positive tissue. In this regard, there were 17 cases (73.9%) with all tissues examined positive.

**Table 3:** Dengue fatal case confirmation by any of the NS1 capture tests per tissues per case.

Fatal Case #	Liver (*n = *23)	Lung (*n* = 14)	Kidney (*n = *4)	Brain (*n = *10)	Heart (*n = *2)	Skin (*n = *1)	Spleen (*n = *15)	Thymus (*n = *3)	Lymph nodes (*n* = 2)	Positive by any NS1 capture test / tissues available per case
**1**	+	−	+	+	NA	NA	−	NA	NA	3/5 (60)
**2**	+	+	NA	+	NA	NA	+	NA	NA	4/4 (100)
**3**	+	NA	NA	NA	NA	+	NA	NA	NA	2/2 (100)
**4**	+	+	NA	NA	NA	NA	NA	NA	NA	2/2 (100)
**5**	+	NA	NA	NA	NA	NA	−	NA	NA	1/2 (50)
**6**	+	+	NA	+	NA	NA	NA	+	−	4/5(80)
**7**	+	+	NA	+	NA	NA	+	+	+	6/6 (100)
**8**	+	+	NA	+	NA	NA	+	NA	NA	4/4 (100)
**9**	+	+	NA	NA	NA	NA	NA	NA	NA	2/2 (100)
**10**	+	+	NA	NA	NA	NA	+	NA	NA	3/3 (100)
**11**	+	NA	+	NA	−	NA	−	NA	NA	2/4 (50)
**12**	+	NA	NA	+	NA	NA	+	+	NA	4/4 (100)
**13**	+	+	NA	−	NA	NA	+	NA	NA	3/4 (75)
**14**	+	NA	NA	NA	NA	NA	NA	NA	NA	1/1 (100)
**15**	+	+	NA	+	NA	NA	+	NA	NA	4/4 (100)
**16**	−	+	NA	+	NA	NA	−	NA	NA	2/4 (50)
**17**	+	NA	NA	NA	NA	NA	+	NA	NA	2/2 (100)
**18**	+	NA	NA	NA	NA	NA	NA	NA	NA	1/1 (100)
**19**	−	NA	NA	NA	NA	NA	NA	NA	NA	0/1
**20**	+	+	+	NA	+	NA	+	NA	NA	5/5 (100)
**21**	+	NA	NA	NA	NA	NA	NA	NA	NA	1/1 (100)
**22**	+	NA	NA	NA	NA	NA	+	NA	NA	2/2 (100)
**23**	+	+	+	+	NA	NA	+	NA	NA	5/5 (100)
**Total** *	**21/23 (91.3)**	**12/13 (92.3)**	**4/4 (100)**	**9/10 (90.0)**	**1/2 (50)**	**1/1 (100)**	**11/15 (73.3)**	**3/3 (100)**	**1/2 (50)**	**63/75 (84)**

*Positive/total analyzed (%), +: positive sample, −: negative sample, NA: not available.

In our study, the Early ELISA test (PanBio) was less efficient in detecting dengue infection in tissues from fatal cases (34.7%) when compared to the Platelia test (60.8%) and the NS1 Ag Strip (91.3%). Specificities were 100% for every NS1 antigen capture tests, based on the three negative tissues for dengue infection in all diagnostic methods available in the Laboratory. No cross-reactivity was observed with tissues from fatal cases of yellow fever.

## Discussion

Dengue diagnosis is based on clinical and laboratory findings. This is of great importance for proper care and treatment of patients, and guide the implementation of measures aimed at the control and prevention of outbreaks and epidemics. Currently, DHF is emerging as an important public health problem in the world, including in the American region and annually a high number of cases are reported [1].

The confirmation of dengue fatal cases has always been troublesome because in most of cases only one blood sample is obtained and the death occurred around defervescence [27] when positive results of expensive and laborious techniques like viral isolation and viral RNA detection [15], [16], [17] might be difficult. The dengue virological diagnosis in tissues specimens is also achieved by experiences personnel by immunohistochemistry [17], [18], [19], [20]. Theses methodologies has allowed the detection of DENV in liver, spleen, brain, lung, lymph node, thymus, kidney, heart, bone marrow and skin [15], [17], [19], [20].

Due to cultural and religious beliefs, the lack of anatomic pathology infrastructure and staff, and biosafety issues, necropsies might not be usually performed, mainly in incoming countries [28]. In the present work, three tests for NS1 antigen capture: Early ELISA test (PanBio), Platelia NS1 (BioRad) and the NS1 Ag Strip (BioRad) were evaluated in tissues of Brazilian fatal dengue cases and antigen detection was 34.7% (8/23), 60.8% (14/23) and 91.3% (21/23), respectively. The Early ELISA detected NS1 in 22.9% (17/74) of the samples evaluated and the Platelia NS1 in 45.9% (34/74). The highest sensitivity was obtained by the NS1 Ag Strip and the differences in the sensitivities were statistically significant (*p<0,05*). Among the ELISA assays studied, the NS1 Ag Strip was the most sensitive in liver, lung, kidney, brain, spleen and thymus. Despite those results, we are not able to infer whether or not this detection was due to in situ viral replication or may be due to the virus present in the blood supporting these tissues.

Only the Real Time RT-PCR technique [22] performed in the evaluated tissues was more sensitive than the NS1 Ag Strip assay. The sensitivities of any of the three NS1 capture assays were higher than the viral isolation and conventional RT-PCR.

Despite a number of prior studies have demonstrated the usefulness of DENV NS1 antigen detection by different ELISA assays in plasma and/or sera of dengue patients [8], [10], [11], [12], [13], [14], no research has been done previously to demonstrate NS1 presence in tissues of fatal dengue. However, in the present work the NS1 antigen was detected in 22 out of 23 dengue fatal cases examined and 73.9% of all tissues specimens evaluated were positive. Most tissues included in this study, liver, lung, kidney, brain, skin, and spleen have been reported with DENV presence in previous studies using molecular and immunohistochemical methods [15], [17], [19], [20], [29]. However, we demonstrated here NS1 in heart and thymus tissue while so far not cardiac tissue has been reported with DENV antigen and/or viral RNA and thymus tissue with DENV RNA has been demonstrated once [30].

The liver was recognized as a major target organ in the pathogenesis of DENV infection, its active hepatocyte replication perhaps accounting for these findings [31], [32]. Furthermore, our findings suggest the liver as the most appropriate tissue for NS1 antigen detection. The breakdown of the blood-brain barrier has been shown previously in fatal dengue cases [19]. In a study of 378 Vietnamese patients with suspected central nervous system infections, 4.2% were infected with DENV [33]. Furthermore, DENV infection could involve the heart and cause cardiac dysfunction, however, lesions in the heart have not been well documented, nevertheless, flame-shaped subendocardial haemorrhage in the left-ventricular septum has been reported [34], cardiac rhythm disorders, such as atrioventricular block [35], [36] and ectopic ventricular beats [37], have been described during episodes of DHF, most of them presenting a benign course with spontaneous resolution. These clinical features have been attributed to viral myocarditis; however the exact mechanism has yet to be elucidated definitively.

Besides common manifestations of dengue infection, thoracic manifestations such as pleural effusion and pneumonitis are described in DHF. Morphological studies of lung tissues revealed interstitial pneumonia associated with focal or diffuse zones of alveolar congestion and hemorrhage, increase of alveolar macrophages number, recruiting of platelets, mononuclear and polymorphonuclear cells [38], [39].Viral antigen was also demonstrated in inflammatory cells of the lung and spleen [39].

The application of NS1 antigen capture kits to demonstrate the presence of DENV may provide a better understanding of viral tropism in fatal cases and may be useful for studies of pathogenesis *in vivo* and in experimental animals. Moreover, NS1 capture ELISAs and ICTs, are rapid, inexpensive and require less laboratory expertise than the molecular and immunohistochemical techniques currently used to detect DENV in tissues.

In fact, the ELISA alternative to confirm DENV infection in suspected dengue fatal cases may be very beneficial in low resources settings facing necropsies rejection due to the small piece of tissue (1–2 g) needed to perform the technique which can be easily obtained via needle biopsy. The needle biopsy has been already proven as a helpful procedure in low resources settings [28] and in dengue studies [17], [40], [41].

The detection of viral antigens in tissues by ELISA has been reported previously in animals, in the European brown hare syndrome virus in hares's splenic tissues [42], West Nile virus antigen in avian tissues [43] and Ebola virus antigen in the spleen and liver tissues from monkeys [44]. Nevertheless, in this report is demonstrated DENV antigen in a number of different human tissues.

In our study, even though we are not able to confirm whether the NS1 antigen captured was from the tissues cells or the circulating blood irrigating those, we aimed here to stress the role of this approach as an alternative tool. However, the presence of DENV antigen in some tissues by immunohistochemistry could infer the presence of NS1 within those tissues. Further immunostaining studies on those tissues by using anti-NS1 antibodies, for instance are suggested to help elucidate those issues. Furthermore, as few negative control samples of tissues other than liver were tested using the NS1 assays, further studies to establish the specificity of this approach are needed before NS1 antigen testing can be relied upon for the diagnosis of fatal dengue.

In summary, even though the lack of common tissues and consistent testing for each tissue for each case may not be the best assessment for this approach, this study demonstrates that DENV NS1 capture assays are a valuable approach to postmortem dengue confirmation and may be used as a clinical/pathological diagnostic tool. To the best of our knowledge, this is the first time an ELISA and an ICT for detecting DENV antigens in tissues is evaluated. The accuracy, sensitivity and rapidity of the NS1 Ag Strip make it suitable for effective dengue surveillance and indicate its use as a complement for the diagnosis of fatal dengue cases. This evaluation was performed for research purposes only and authors have no financial interest.
